# Serum albumin and derived neutrophil-to-lymphocyte ratio are potential predictive biomarkers for immune checkpoint inhibitors in small cell lung cancer

**DOI:** 10.3389/fimmu.2024.1327449

**Published:** 2024-06-07

**Authors:** Zhanpeng Kuang, Jessica Miao, Xiaoli Zhang

**Affiliations:** ^1^College of Public Health, The Ohio State University, Columbus, OH, United States; ^2^College of Arts and Sciences, The Ohio State University, Columbus, OH, United States; ^3^Department of Biomedical Informatics, The Ohio State University, Columbus, OH, United States

**Keywords:** SCLC, immune checkpoint inhibitors, albumin, derived neutrophil to lymphocyte ratio, response, overall survival, prognostic scoring system

## Abstract

**Background:**

Immune checkpoint inhibitors (ICIs) have reshaped the treatment landscape of small cell lung cancer (SCLC), but only a minority of patients benefit from this therapy. Therefore, it is critical to identify potential risk factors that could predict the efficacy of ICI treatment in SCLC patients and identify patient subgroups who may benefit the most from ICI therapy.

**Methods:**

Our study included a total of 183 SCLC patients who had received at least one dose of ICI treatment. We utilized both logistic regression and Cox proportional hazard regression to evaluate whether various patient clinical factors and serum biomarkers could serve as predictors of patient response to treatment and overall survival (OS) during ICI therapy.

**Results:**

Logistic regression showed that patients with a history of surgery (p=0.003, OR 9.06, 95% CI: (2.17, 37.9)) and no metastasis (p=0.008, OR 7.82, 95% CI: (1.73, 35.4)) exhibited a higher odds of response to ICI treatment. Cox regression analyses demonstrated that pretreatment blood albumin (p=0.003, HR 1.72, 95% CI: (1.21, 2.45)) and derived neutrophil to lymphocyte ratio (dNLR) (p=0.003, HR 1.71, 95% CI: (1.20–2.44)) were independent predictors for OS in SCLC patients. By establishing a pre-treatment prognostic scoring system based on baseline albumin and dNLR, we found that patients with high albumin and low dNLR exhibited a significantly better prognosis than those with low albumin and high dNLR in both the full (P<.0001, HR 0.33, 95% CI: 0.20–0.55) and the metastatic cohort (P<.0001, HR 0.28, 95% CI: 0.15–0.51). The better prognostic group also had younger age, higher BMI and lower systemic inflammatory biomarker values than the unfavorable group (P<.0001).

**Conclusion:**

Our data reveals the significant role of metastasis status and treatment history in predicting the initial response of SCLC patients to ICI treatment. However, baseline serum albumin and dNLR provide a more precise prognostic prediction for patient OS. The scoring system based on albumin and dNLR enhances the ability to stratify patient prognosis and holds the potential to guide clinical decision-making for SCLC patients undergoing ICI therapy.

## Introduction

Small cell lung cancer (SCLC) is an exceptionally aggressive malignancy characterized by early metastasis and rapid tumor growth, resulting in a discouraging 5-year overall survival rate ranging only from 1% to 5% (1, 2). SCLC represents approximately 13–15% of total lung cancer cases (3) and results in between 25,000–30,000 deaths annually in the United States. SCLC is categorized into two primary stages based on the staging system: limited-disease SCLC (LD-SCLC) and extensive-disease SCLC (ED-SCLC). Majority of patients, around two-thirds, are diagnosed with extensive stage or metastatic disease at the time of diagnosis (4). In contrast to non-small cell lung cancer (NSCLC), which benefits from a wide array of treatment options, including targeted agents and immunotherapies (5–9), the treatment landscape for SCLC has seen little evolution over the past few decades, while platinum-based chemotherapy in combination with etoposide remains the standard of care for SCLC (10).

Immunotherapy, particularly immune checkpoint inhibitors (ICIs), have revolutionized cancer treatment, yielding increased response rates and prolonged survival for a wide range of cancer types. By targeting key immune checkpoint molecules like programmed cell death protein 1 (PD-1), programmed cell death ligand 1 (PD-L1), and cytotoxic T lymphocyte-associated antigen 4 (CTLA-4), ICIs have the capacity to stimulate T cells and activate the immune system, enabling it to target and kill cancer cells (11). In the case of SCLC, the recent introduction of ICIs has reshaped its treatment landscape. Notably, the combination of anti-PD-L1 antibody durvalumab and atezolizumab with platinum-based chemotherapy (either carboplatin or cisplatin plus etoposide) has emerged as a first line treatment for patients with extensive stage SCLC, exhibiting significantly improved overall survival (12–15). However, the efficacy of immunotherapy remains limited, benefiting only a small subset of SCLC patients (16–19). Hence, it is critical to identify factors and biomarkers predicting the efficacy of ICI therapy in SCLC patients and identify SCLC patients who may benefit from ICI treatment.

Recent studies have explored various potential risk factors for predicting the effectiveness of ICI treatment. Tumor mutational burden (TMB) (20) and PD-L1 positivity (21) are among the most studied (22, 23). Additionally, factors such as metastasis status (24, 25) and several serum-based biomarkers, including systemic inflammatory biomarkers like neutrophil to lymphocyte ratio (NLR) (23, 26, 27) and derived NLR (28), albumin levels (29, 30), platelet to lymphocyte ratio (23, 31), lactate dehydrogenase (LDH) (32–34), Lung Immune Prognostic Index that integrates dNLR and LDH (23, 35, 36), have shown promise as prognostic indicators for ICI efficacy (37). However, it’s important to note that increased TMB and high PD-L1 expression do not consistently result in better treatment responses (17, 20, 22, 38). Additionally, other biomarkers have also produced conflicting results, underscoring the necessity for further research to validate their predictive roles (34, 37). Furthermore, given the lack of prior assessment of these factors in SCLC patients who receive ICI-involved immunochemotherapy, it is of particular importance to investigate these biomarkers in predicting ICI outcomes in SCLC. This research holds significant potential to aid in the development of more precise and effective treatment strategies for SCLC patients.

In this study, we present the results of a retrospective SCLC cohort data analysis conducted at a single institution. This analysis investigates the impact of baseline clinical factors and various serum inflammatory biomarkers, on the effectiveness of ICI treatment in patients with SCLC. Our primary objective is to contribute to the identification of specific patient subgroups that are best suited for ICI treatment. We believe that these findings can offer valuable guidance for clinical decision-making and ultimately lead to the improvement of patient care.

## Methods

### Study cohort and patient clinical information

The clinical data of SCLC patients were collected by Pelotonia Institute for Immuno-Oncology (PIIO) at The Ohio State University (OSU) Wexner Medical Center under approved Institutional Review Board (IRB) protocol IRB 2020C0145. The data gathered retrospectively from the Clarity electronic medical record database, which stores Epic/IHIS data of patients. The study included a total of 183 SCLC patients who received at least one dose of immune checkpoint inhibitor treatment (which included anti-PD1 inhibitors: Pembrolizumab, Nivolumab, Cemiplimab; anti-PD-L1 inhibitors: Atezolizumab, Durvalumab, and Avelumab; and anti-CTLA4 inhibitors: Ipilimumab) between November 2011 and December 2021. The patient inclusion criteria required that patients needed to be over 18 years of age at enrollment, had only one cancer diagnosis, received treatment within the study period, and had available overall survival data.

The patient clinical information and categorization of each variable are provided as a codebook in **Supplementary Data 1**. The patient baseline characteristics are summarized in **Supplementary Table S1**. The median age was 64 (range: 32–91) years, and the median Body Mass Index (BMI) was 25.3 (range: 13.7–56.0) kg/m2. There was a higher representation of males (56.3%) than females. Majority of patients were white (90.2%), married (57.4%), and retired (54.6%).

Among all the patients who had clinical stage information, 43.1% (59 out of 137) had advanced tumor stage, while 78.2% (129 out of 165) had advanced N stage, and 76.8% (129 out of 168) had metastasis. All 183 patients received a combination of ICI and chemotherapy. Regarding the ICI type, 168 patients (91.8%) received anti-PD-1/PD-L1 inhibitor therapy, and 15 patients received a combination of anti-CTLA4 inhibitor (ipilimumab) and anit-PD1 inhibitor (nivolumab) treatment. Only 31 (16.9%) underwent surgery, and 70 (38.3%) received radiation treatment among all the patients.

Baseline blood was taken before treatment, and hematological markers included serum lactate dehydrogenase (LDH) (U/L), albumin (Alb) (g/L), eosinophil, neutrophil to lymphocyte ratio (NLR), derived neutrophil to lymphocyte ratio (dNLR= ANC/(WBC−ANC)), platelet to lymphocyte ratio (PLR), lymphocyte to monocyte ratio (LMR), systemic inflammation index (SII=Platelet counts x neutrophil counts/lymphocyte counts), and systemic inflammation response index (SIRI= neutrophil count × monocyte count/lymphocyte count). These hematological biomarkers were calculated as previously described (39, 40), and the data was dichotomized based on the median value (albumin, NLR, dNLR, PLR, LMR, SII, and SIRI) or upper limit of normal range (LDH>190 g/L as used in OSU medical center) (**Supplementary Data 1**). Levels of individual blood cells including neutrophils, WBC, lymphocytes, monocytes, and platelets were used for univariate analysis to test their association with patient survival but were not in the multivariate model even if the cell type was significantly associated with survival. This is to avoid repeated use of the same value and collinearity of variables as the systemic inflammatory biomarkers (NLR, dNLR, PLR, LMR, SII and SIRI) were calculated from these individual values.

### Outcomes

This study focused on patient complete response and overall survival (OS). The data recorded patients’ first recurrence as “no recurrence”, “recurred”, and “never disease free” with date of recurrence defined as the date of radiographic detected disease. For response endpoint, we considered patients with “no recurrence” and “recurred” as those who were responders with complete response and others as non-responders. The OS time was defined as the time between the earliest ICI ordering and death for any reason or the end of the study period.

### Statistical analyses

Continuous variables were analyzed with two-sample t-test or ANOVA. The hematological variables were first log transformed to reduce skewness and variance before comparison. Categorical variables were analyzed with Chi-square test or Fisher’s exact test. Univariate and multivariate logistic regression was used to test the association of risk factors with patient response. Log rank test with Kaplan-Meier curves, univariate and multivariate Cox proportional hazard regression was performed to evaluate the association between the listed factors and overall survival. Since missing values are a common issue for observational data, our data also have different levels of missingness for different variables. Therefore, for univariate analyses, we used the available values for each variable. For multivariate analyses, we used both complete data and imputed data. Variables with p < 0.05 in univariate analysis were included in the logistic regression or multivariate Cox proportional-hazards model and then backward selected until no further variables could be eliminated based on model fit (p < 0.05). Tests were considered statistically significant with a two-sided P-value of ≤0.05. SAS 9.4 and R-studio were used for analyses and making figures.

### Prognostic scoring system development

For the development of a predictive scoring system for overall survival using pretreatment serum albumin and dNLR level, both variables were dichotomized into low (<median) and high (≥median) based on the individual median value. Patients with a low dNLR and high albumin levels received a score of 2 and were assigned into the “good prognosis group.” Patients with high dNLR and low albumin levels were assigned a score of 0, designating them as the “bad prognosis group.” Patients with either both high or both low levels of pretreatment albumin and dNLR received a score of 1 and were categorized as the “intermediate group”. Then the association between the different prognostic groups and OS was tested using Log-rank test, and further validated using k (k=3)-fold cross validation.

## Results

### Patient laboratory values

In this study, over 97% of patients had documented pre-treatment laboratory results. **Table 1** and **Supplementary Table S2** provides a summary of the median and interquartile range (IQR) for the available lab values. However, it’s important to note that LDH had a very high percentage of missing data (74.9%), whereas the remaining laboratory values had missingness ranging from 2.7% to 7.1%. For our multivariate model, all the selected lab biomarkers were imputed for separate analysis to ensure a more accurate examination.

**Table 1:** Patient baseline hematological biomarkers for the full cohort and the metastatic subgroup.

Hematological Biomarkers	Total SCLC Median (IQR)	Total SCLC n (%)	ED-SCLC Median (IQR)	ED-SCLC n (%)	Total Missing n (%)
**LDH (U/L)**	216 (159, 295)	46	236 (175, 311)	25	137 (74.9%)
High		27 (58.7%)		16 (64.0%)	
Low		19 (41.3%)		9 (36.0%)	
**Alb (g/dL)**	3.7 (3.5, 4.0)	176	3.7 (3.4, 4.0)	124	7 (3.8%)
High		86 (48.9%)		54 (43.5%)	
Low		90 (51.1%)		70 (56.5%)	
**eosinophil**	0.1(0.05, 0.19)	132	0.08 (0.04, 0.18)	92	51 (27.9%)
High		67 (50.8%)		42 (45.7%)	
Low		65 (49.2%)		50 (54.3%)	
**NLR**	4.8 (2.7, 7.9)	170	4.7 (3.0, 7.7)	123	13 (7.1%)
High		85 (50.0%)		61 (49.6%)	
Low		85 (50.0%)		62 (50.4%)	
**dNLR**	2.5 (1.7, 3.5)	170	2.5 (1.8, 3.4)	123	13 (7.1%)
High		85 (50.0%)		62 (50.4%)	
Low		85 (50.0%)		61 (49.6%)	
**PLR**	244 (143, 365)	178	223 (131, 361)	126	5 (2.7%)
High		89 (50.0%)		62 (49.2%)	
Low		89 (50.0%)		64 (50.8%)	
**LMR**	1.7 (1.0, 2.6)	178	1.7 (1.1, 2.6)	126	5 (2.7%)
High		89 (50.0%)		63 (50.0%)	
Low		89 (50.0%)		63 (50.0%)	
**SII**	1402 (639, 2595)	170	1391 (633, 2644)	123	13 (7.1%)
High		85 (50.0%)		61 (49.6%)	
Low		85 (50.0%)		62 (50.4%)	
**SIRI**	3.4 (1.7, 6.9)	170	3.4 (1.7, 6.9)	123	13 (7.1%)
High		85 (50.0%)		62 (50.4%)	
Low		85 (50.0%)		61 (49.6%)	

### Patient response

We first compared the clinical characteristics between responders and non-responders, as illustrated in **Supplementary Table S3**. Among all SCLC patients, only 15 out of 183 patients were considered as responders with complete response (8.2%), with 7 experiencing a later recurrence. Univariate logistic regression revealed that patients who underwent surgery (p<0.001), had a lower N stage (p<0.001), and had no metastasis (p<0.001) had significantly higher odds of response (**Table 2**). None of the blood biomarkers were significantly associated with patient response to ICI treatment. Multivariate analyses demonstrated that undergoing surgery (p=0.003, OR=9.06 (2.17, 37.86)) and absence of metastasis (p=0.008, OR=7.82 (1.73, 35.43)) were the independent variables associated with higher odds of response to ICI treatment (**Table 2**).

**Table 2:** Logistic regression analyses results showing the association of clinical factors with response to ICI therapy in SCLC patients (including only the variables with P<0.1 from univariate analysis).

Clinical Characteristic	Univariate logistic regression		Multivariate logistic regression	
	OR (95% CI)	p-value	OR (95% CI)	p-value
Clinical N Stage				
High Stage	—		—	
Low Stage	8.93 (2.51, 31.74)	**<0.001**	3.65 (0.85, 15.65)	**0.08**
Clinical M Stage				
Metastasis	—		—	
No Metastasis	14.48 (3.75, 55.97)	**<0.001**	7.82 (1.73, 35.43)	**0.008**
Radiation				
No	—			
Yes	0.22 (0.05, 1.02)	0.054		
Surgery				
No				
Yes	13.91 (4.33, 44.66)	**<0.001**	9.06 (2.17, 37.86)	**0.003**

### Overall survival analysis of all patients

**Figure 1:**
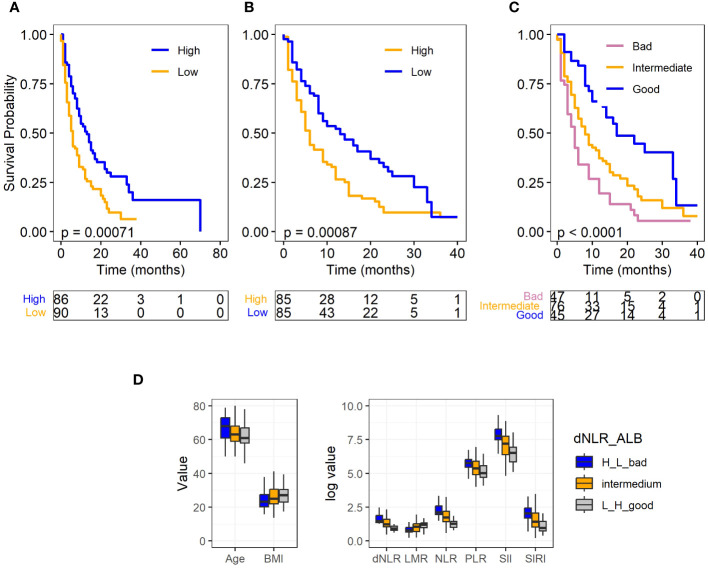
Survival plots for full SCLC cohort and levels of clinical factors in different prognostic groups. **(A)** Albumin level and OS. **(B)** dNLR level and OS. **(C)** Different prognostic groups and OS. **(D)** Levels of clinical factors including age, BMI, and hematological biomarkers in different prognostic groups. Albumin and dNLR was dichotomized to high and low according to their median values. Log-rank test was used for the analysis for the OS, and ANOVA was used for the comparison of the clinical factors and hematological biomarkers among different prognostic groups.

By the end of the study, 40 patients (21.9%) were still alive. The median survival of all patients was 8 (95%CI: 9.4,12.5) months. In the univariate analyses we found that patients with older age (≥64 years) (p=0.039, HR=1.42, 95%CI:1.02, 1.97), low albumin (≤3.7g/dL) (p<0.001, HR=1.80, 95%CI:1.28, 2.54), high NLR (>4.8) (p=0.006, HR=1.63, 95%CI:1.15, 2.31), and high dNLR (>2.5) (p=0.001, HR=1.76, 95%CI:1.24, 2.50) had significantly increased risk for worse OS (**Table 3**; **Supplementary Table S4**). Subsequently, based on the complete data (N=168, event=128), low blood albumin levels (p=0.003, HR 1.72, 95% CI: 1.21–2.45) and high dNLR (p=0.003, HR 1.71, 95% CI: 1.20–2.44) were identified as the independent risk factors predicting worse OS for SCLC in multivariate analysis. **Figures 1A, B** display the association of albumin and dNLR with OS, respectively, based on the Log-rank tests. Imputed data analyses with all 183 patients also showed similar results (**Supplementary Data 2**), where albumin and dNLR were the only independent predictors for OS.

**Table 3:** Full patient cohort univariate and multivariate Cox proportional hazard regression model results.

Clinical	Univariate				Multivariate	
Characteristics	Total	Event	HR (95% CI)*^1^*	p-value	HR (95% CI)*^1^*	p-value
**Age**	183	143				
≥ 64			1.42 (1.02 to 1.97)	**0.039**	1.28 (0.90 to 1.83)	0.16
< 64			—		—	
**Sex**	183	143				
Female			—			
Male			1.29 (0.93 to 1.80)	0.13		
**Race**	183	143				
African American/Black			—			
White			1.75 (0.92 to 3.34)	0.065		
**BMI**	183	143				
≥ 25			—			
< 25			1.23 (0.89 to 1.72)	0.21		
**Marital Status**	176	140				
Married			—	0.11		
Other			0.70 (0.45 to 1.08)			
Single			0.69 (0.45 to 1.07)			
**Employment Status**	183	143				
Employed			—	0.11		
Not Employed			0.60 (0.37 to 0.99)			
Retired			0.83 (0.54 to 1.29)			
**Clinical T Stage**	137	110				
High Stage			—			
Low Stage			0.97 (0.66 to 1.41)	0.87		
**Clinical N Stage**	165	131				
High Stage			—			
Low Stage			0.74 (0.48 to 1.15)	0.17		
**Clinical M Stage**	168	134				
Metastasis			—			
No Metastasis			0.75 (0.50 to 1.13)	0.16		
**ICI Type**	183	143				
Anti-PD1/PD-L1 Inhibitor			0.65 (0.37 to 1.16)	0.15		
Combination ICI			—			
**Radiation**	183	143				
No			—			
Yes			0.85 (0.60 to 1.19)	0.34		
**Surgery**	183	143				
No			—			
Yes			0.69 (0.44 to 1.07)	0.087		
**LDH (U/L)**	46	38				
High			0.97 (0.50 to 1.89)	0.92		
Low			—			
**Albumin (g/dL)**	176	136				
High			—		—	
Low			1.80 (1.28 to 2.54)	**<0.001**	1.72 (1.21 to 2.45)	**0.003**
**Eosinophils**	132	105				
High			0.87 (0.60, 1.30)	0.54		
Low						
**NLR**	170	130				
High			1.63 (1.15 to 2.31)	**0.006**	1.03 (0.67 to 1.57)	0.91
Low			—		—	
**dNLR**	170	130				
High			1.76 (1.24 to 2.50)	**0.001**	1.71 (1.20 to 2.44)	**0.003**
Low			—		—	
**PLR**	178	138				
High			1.08 (0.77 to 1.51)	0.65		
Low			—			
**LMR**	178	138				
High			1.09 (0.78 to 1.53)	0.61		
Low			—			
**SII**	170	130				
High			1.24 (0.88 to 1.74)	0.23		
Low			—			
**SIRI**	170	130				
High			1.32 (0.93 to 1.87)	0.12		
Low			—			

^1^HR, Hazard Ratio; CI, Confidence Interval.

### Prognostic scoring for full SCLC cohort

We proceeded to develop a prognostic scoring system that incorporates pretreatment serum albumin level and dNLR, and classified patients into three different prognostic groups. Patients with a low dNLR and high albumin levels received a score of 2 and were assigned into the “good prognosis group.” Conversely, those with high dNLR and low albumin levels were assigned a score of 0, designating them as the “bad prognosis group.” The rest of patients received a score of 1, placing them in the “intermediate group.” This group includes patients with either both high or both low levels of pretreatment albumin and dNLR.

Out of the 168 patients with complete data, 45 were categorized into the “good prognosis group,” 76 into the “intermediate group,” and 47 into the “bad prognosis group.” The median survival times for these groups were 14.91 months, 10.94 months, and 7.51 months, respectively. As demonstrated in **Figure 1C**, the log-rank test revealed a statistically significant difference in overall survival (P<.0001) among these three groups. The Cox model further confirmed that the good (p<.0001, HR: 0.33, 95% CI: 0.20–0.55) and intermediate groups (p=0.03, HR: 0.65, 95% CI: 0.44–0.96) exhibited a significantly reduced risk of death in comparison to the bad prognosis group. Furthermore, to test the robustness of our prognostic biomarker, a combination of dNLR and serum albumin level, we conducted an internal 3-fold cross validation. Despite each fold comprising only 33.3% of patient samples (n=56), our results demonstrated the stability of the prognostic power of the scoring system across different subsets of the data. The prognostic biomarker remained significantly associated with patient overall survival, with notable separation observed between the “good” and “bad” prognostic groups (**Supplementary Figure S1**).

Previous research has indicated that factors such as age, BMI and systemic inflammatory biomarkers are associated with patient responses to immunotherapy. To examine whether these variables are correlated with the prognostic groups, we conducted a comparison among the different prognostic groups using ANOVA. The results demonstrated that the good prognostic group had significantly younger age, higher BMI, and different level of systemic inflammatory biomarkers, including decreased level of PLR, NLR, SIRI, SII and increased level of LMR (P<.0001). Furthermore, as expected, this group also had higher albumin levels and lower dNLR (P<.0001) (**Figure 1D**). These findings reinforce the role of these factors in predicting patient outcomes following immunotherapy.

### Subgroup OS analysis for metastatic patients

Considering that majority of SCLC patients are diagnosed at the extensive stage, we conducted a subgroup analyses of risk factors that could predict OS in individuals with advanced metastatic diseases (ED-SCLC cohort). In this subgroup study, 129 SCLC patients who presented with metastasis were included. Among them, 25 were alive at the end of the study. The median survival for this cohort was 6 months (95% CI: 8–11 months).

**Figure 2:**
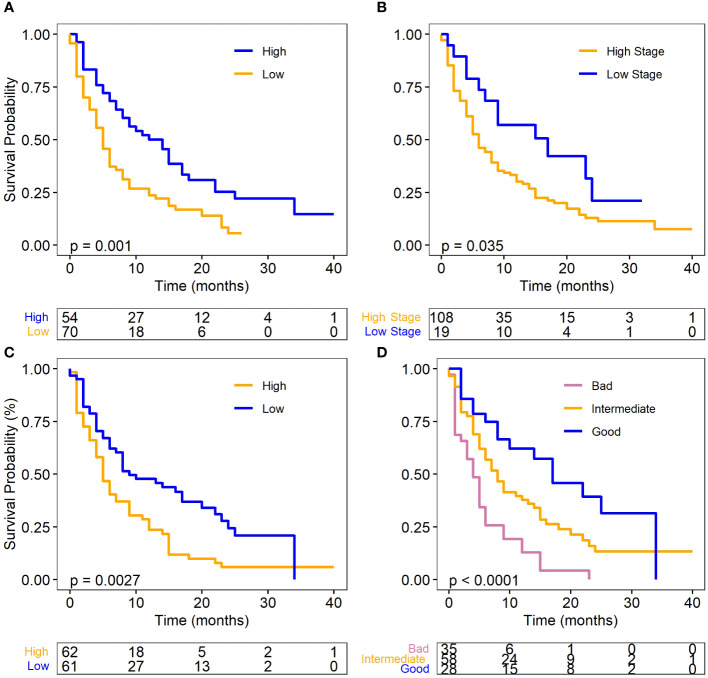
Survival plots for metastatic SCLC (ED-SCLC) cohort. **(A)** Albumin level and OS. **(B)** dNLR level and OS. **(C)** N stage and OS. **(D)** Different prognostic groups and OS. Log-rank test was used for the analysis for the OS. Albumin and dNLR was dichotomized to high and low according to their median values.

For this patient cohort, the results mirrored those of the overall cohort analysis (**Table 4**), which included patients with and without metastasis. Univariate Cox regression analysis revealed that low albumin levels (p=0.002, HR: 1.92, 95% CI: 1.27–2.91), high dNLR (p=0.005, HR: 1.80, 95% CI: 1.20–2.69), and high N-stage (p=0.046, HR: 0.85, 95% CI: 1.01–3.39), were significantly associated with an increased risk of death. The results were displayed with Kaplan-Meier survival curves as shown in **Figure 2**.

**Table 4:** Metastatic patient cohort univariate and multivariate Cox proportional hazard regression model results.

ED_SCLC Clinical	Univariate				Multivariate	
Characteristic	N	Event N	HR (95% CI)*^1^*	p-value	HR (95% CI)*^1^*	p-value
**Age**	129	104				
≥ 64			1.24 (0.84 to 1.82)	0.27		
< 64			—			
**Sex**	129	104				
Female			—			
Male			1.13 (0.76 to 1.68)	0.54		
**Race**	129	104				
African American/Black			—			
White			1.90 (0.88 to 4.10)	0.073		
**BMI**	129	104				
≥ 25			—			
< 25			1.28 (0.87 to 1.88)	0.21		
**Marital Status**	122	101				
Married			—	0.61		
Other			1.06 (0.64 to 1.78)			
Single			0.80 (0.48 to 1.33)			
**Employment Status**	129	104				
Employed			—	0.41		
Not Employed			0.69 (0.39 to 1.23)			
Retired			0.87 (0.52 to 1.45)			
**Clinical T Stage**	101	81				
High Stage			—			
Low Stage			0.92 (0.59 to 1.43)	0.72		
**Clinical N Stage**	127	102				
High Stage			1.85 (1.01 to 3.39)	**0.046**	2.21 (1.16 to 4.19)	**0.015**
Low Stage			—		—	
**ICI Type**	129	104				
Anti-PD1/PD-L1 Inhibitor			0.66 (0.35 to 1.24)	0.20		
Combination ICI			—			
**Radiation**	129	104				
No			—			
Yes			0.86 (0.58 to 1.29)	0.47		
**Surgery**	129	104				
No			—			
Yes			0.57 (0.31 to 1.05)	0.054		
**LDH (U/L)**	25	22				
High			1.44 (0.60 to 3.46)	0.41		
Low			—			
**Albumin (g/dL)**	124	99				
High			—		—	
Low			1.92 (1.27 to 2.91)	**0.002**	2.18 (1.42 to 3.35)	**<0.001**
**Eosinophils**	92	77				
High			1.07 (0.68, 1.68)	0.76		
Low			—			
**NLR**	123	98				
High			1.54 (1.03 to 2.30)	**0.035**	1.05 (0.58, 1.93)	0.86
Low			—		—	
**dNLR**	123	98				
High			1.80 (1.20 to 2.70)	**0.005**	1.91 (1.26 to 2.91)	**0.002**
Low			—		—	
**PLR**	126	101				
High			1.20 (0.81 to 1.78)	0.35		
Low			—			
**LMR**	126	101				
High			0.81 (0.55 to 1.19)	0.29		
Low			—			
**SII**	123	98				
High			1.29 (0.87 to 1.93)	0.20		
Low			—			
**SIRI**	123	98				
High			1.36 (0.91 to 2.03)	0.13		
Low			—			

^1^HR, Hazard Ratio; CI, Confidence Interval.

In the multivariate analysis, all three factors were identified as independent predictors of OS in the metastatic cohort (complete data: n=121, with 95 events). A decrease in albumin levels (p=0.0004, HR: 2.18, 95% CI: 1.42–3.35), an increase in N-stage (p=0.015, HR: 2.21, 95% CI: 1.16–4.19), and a higher dNLR (p=0.002, HR: 1.91, 95% CI: 1.26–2.91) were associated with an increased risk of death (**Table 4**). Moreover, the analysis of imputed data (n=129, with 104 events) also identified these three variables as independent factors predicting OS (**Supplementary Data 3**).

Similar to the full cohort, the prognostic scoring system also categorized the ED-SCLC patients into “good (n=28),” “intermediate (n=58),” and “bad (n=35)” groups. The median survival was 11, 8, and 4 months for the good, intermediate, and bad group respectively. The good prognostic group had significantly better OS than the bad prognostic group (P<.0001, HR=0.28, 95%CI: 0.15, 0.51) (**Figure 2D**). These findings further highlight the importance of these biomarkers in predicting SCLC patient outcomes in the context of ICI therapy.

## Discussion

In our current study, we performed a comprehensive retrospective analysis on patient baseline risk factors to predict ICI treatment efficacy in SCLC. We placed a specific emphasis on evaluating a series of hematological biomarkers, including LDH, albumin, NLR, dNLR, PLR, LMR, SII, SIRI, as well as individual blood cell types eosinophils, neutrophils, WBC, lymphocytes, monocytes, and platelets on their significance in predicting the effectiveness of ICI treatment in SCLC. It’s noteworthy that such an investigation has not been previously explored in SCLC patients who have undergone ICI involved immunochemotherapy.

Through our study, we first highlighted the significance of the pretreatment blood albumin level as an independent prognostic marker for overall survival in SCLC patients with ICI treatment. The results demonstrated that patients with pretreatment low albumin levels (≤3.7 g/dL) exhibited significantly worse overall survival. This finding holds true not only in the entire SCLC patient cohort (p=0.003, HR 1.72, 95% CI: 1.21–2.45) but also in the subgroup analysis focused on the metastatic patients (p=0.0004, HR: 2.18, 95% CI: 1.42–3.35). Serum albumin has been widely used in assessing patients’ general nutritional health (41) and as a prognostic biomarker for cancer (30, 42, 43). Recently it has emerged as a potential biomarker for predicting the efficacy of ICI therapy (29, 44). In NSCLC, serum albumin has been identified as a potential biomarker for assessing the efficacy of ICI treatment alone or ICI involved immunochemotherapy, either alone or in combination with other markers (29, 40, 45–48). A recent pan-cancer study indicated that in SCLC, patients with elevated albumin levels had improved response and survival outcomes with ICI treatment (22). They determined an optimal cutoff of albumin >3.7 g/dL across different cancer types, and this same cutoff was utilized in our study. With a larger patient cohort, our study has further confirmed the significance of pretreatment serum albumin as a prognostic indicator for predicting the survival outcomes of SCLC patients undergoing ICI involved immunochemotherapy.

A second noteworthy finding in our study is the identification of the derived neutrophil-to-lymphocyte ratio (dNLR) at baseline as another independent risk predictor for the effectiveness of ICI treatment. In contrast to albumin, elevated dNLR level was associated with an increased risk of death, both in the overall SCLC patient cohort (p=0.003, HR 1.71, 95% CI: 1.20–2.44) and in those with ED-SCLC (p=0.002, HR: 1.91, 95% CI: 1.26–2.91). Both NLR and dNLR serve as indicators of systemic inflammation and are widely utilized as prognostic factors in predicting mortality, not only in the general population (28) but also across various disease types (23, 29). This includes a range of solid cancers such as colorectal cancer and melanoma, in both the broader cancer patient population and specifically among those receiving immunotherapy (30–32). Recently, low dNLR was reported to be associated with better outcomes in NSCLC patients who received ICI therapy (28, 29). However, to our best knowledge, our study is the first to demonstrate the critical prognostic significance of dNLR in predicting ICI treatment outcomes in SCLC.

Previous studies have shown that lung immune prognostic index (LIPI), a marker that combines dNLR and LDH, is a prognostic factor of ICI response for NSCLC and other solid tumors (36, 49, 50) as well as for survival outcomes of SCLC patients who received chemotherapy (39). However, due to the great missingness (~ 70%) of LDH values, we could not test the predictive power of LIPI in predicting ICI response in our SCLC cohort. In contrast, we developed a scoring system to evaluate the combined effects of pretreatment serum albumin and dNLR in predicting patient outcomes and identifying specific patient subgroups. For the full patient cohort, patients with a high dNLR and low albumin received a score of 0, and they experienced significantly worse overall survival compared to those with a low dNLR and high albumin who received a score of 2 (P<.0001). The median survival for the group with a favorable prognosis (score=2) was 14.91 months, whereas it was 7.51 months for the group with an unfavorable prognosis (score=0) (HR: 0.33, 95% CI: 0.20–0.55). Notably, the intermediate group, which included patients with either both high or both low albumin and dNLR (score=1), also exhibited significantly better survival than the unfavorable prognosis group (p=0.03, HR: 0.65, 95% CI: 0.44–0.96) but worse than the favorable group (p=0.005, HR: 0.1.98, 95% CI: 1.23–3.17), with a median survival of 10.94 months. To test the robustness of this prognostic scoring system, we did a 3-fold internal cross-validation. The results showed that the scoring system was significantly correlated with patient OS with great separation of the good from the bad prognostic group even with only 33.3% of patient samples (n=56), demonstrating the stability of the findings across different subsets of the data. This scoring system also effectively identified three distinct prognostic groups in patients with ED-SCLC, leading to significantly different survival outcomes (P<.0001). Again, the favorable prognosis group had significantly better survival than the unfavorable group (P<.0001, HR 0.28, 95% CI: 0.15–0.51) with a median survival of 11 months vs. 4 months. Furthermore, the good prognosis group had significantly younger age, higher BMI, and different levels of systemic inflammatory biomarkers, including significantly lower levels of PLR, NLR, SIRI, SII, higher LMR when compared to the unfavorable group. Although the mechanism remains unclear, these systemic inflammatory biomarkers have been reported to be associated with patient survival outcomes and responses to immunotherapy across various cancer types and are now considered as potential prognostic biomarkers (51–54). Our findings confirmed the relevance of these factors with patient outcomes following ICI therapy in the context of SCLC, even though they were not found to be independent factors in predicting patient survival. In summary, the developed scoring system serves as a valuable tool for stratifying SCLC patients into different prognostic categories. This approach has the potential to significantly enhance personalized clinical decision-making and improve the management of SCLC patients, ultimately leading to better patient care and outcomes. However, the validation of this scoring system and comparison with LIPI in predicting ICI response in SCLC still needs to be established in future studies with large independent data.

In addition to our main findings, we also observed that the metastasis status and a patient’s surgical history were significantly associated with the initial response to ICI treatment, but not necessary with patient OS, which is in line with previous findings (27). This is not surprising as patients without metastasis and/or with a history of surgery were likely at an early stage of their disease compared to others and were likely to have better response to the treatment. These factors should be considered in the overall treatment approach for SCLC patients. Due to the limited (n=15) cases as responders based on their first recurrence data, primarily due to the absence of information on partial response and stable disease, it is imperative for further research with a larger sample size to assess the impact of other clinical factors besides metastasis and surgical history on the response to ICI treatment in SCLC patients.

In summary, our study emphasizes the significance of pretreatment serum albumin and dNLR levels as significant potential predictors for patient survival in SCLC patients undergoing ICI therapy. Utilizing the combination of these markers can enhance the identification of patient subgroups most likely to benefit from ICI treatment. This contributes to the potential for more effective and personalized treatment strategies for SCLC patients.

## Conclusion

To our knowledge, our study stands as the first to conduct an extensive examination of hematological biomarkers for predicting the effectiveness of ICIs in SCLC. Although our study was limited to the data from a single center and with missing values and variables that are critical for a more complete assessment, our findings highlight the importance of baseline serum albumin level and dNLR in predicting the overall survival of SCLC patients undergoing ICI-involved immunochemotherapy. They also shed light on the potential subgroups that may benefit from ICI therapy. Additionally, our study underscores the importance of factoring in clinical stages and treatment history when predicting initial patient responses. However, the translation of these results into practical guidance for clinical practices should be further evaluated through larger multicenter studies. This step is essential for confirming the utility of these biomarkers in the management of SCLC patients receiving ICI treatment.

## Data availability statement

The data analyzed in this study is subject to the following licenses/restrictions: These are internally collected patient electronic medical records based on an approved Institutional Review Board (IRB) protocol IRB 2020C0145). Requests to access these datasets should be directed to the corresponding author (xiaoli.zhang@osumc.edu).

## Ethics statement

The studies involving humans were approved by Institutional Review Board (IRB) protocol IRB 2020C0145. The studies were conducted in accordance with the local legislation and institutional requirements. The participants provided their written informed consent to participate in this study.

## Author contributions

ZK: Formal analysis, Writing – review & editing, Data curation, Visualization. JM: Formal analysis, Writing – review & editing. XZ: Formal analysis, Writing – review & editing, Conceptualization, Funding acquisition, Investigation, Methodology, Supervision, Validation, Writing – original draft.

## Supplementary material

Supplementary Figure 1 3-fold cross validation validates the prediction power of the dNLR-Albumin scoring system for patient overall survival displayed with Kaplan-Meier survival curves.

Supplementary Table 1 Patient Clinical Characteristics.

Supplementary Table 2 Summary statistics of patient baseline individual hematological cell types for the full cohort and the metastatic subgroup.

Supplementary Table 3 Logistic regression results showing the association of clinical factors with patient response to ICI therapy in SCLC.

Supplementary Table 4 Univariate Cox proportional hazard regression testing the association of each individual cell types of hematological biomarkers and SCLC patient OS.
