# Supplementary material for: Serum albumin and derived neutrophil-to-lymphocyte ratio are potential predictive biomarkers for immune checkpoint inhibitors in small cell lung cancer
Source: Front Immunol. 2024 Jun 7;15:1327449. doi: 10.3389/fimmu.2024.1327449 (PMC11190784; doi:10.3389/fimmu.2024.1327449)
Supplement: Supplementary Figure 1 — 3-fold cross validation validates the prediction power of the dNLR-Albumin scoring system for patient overall survival displayed with Kaplan-Meier survival curves. [file Image_1.pdf]

Figure S1. 3-fold cross validation validates the prediction power of the dNLR-Albumin scoring system for patient overall survival displayed with Kaplan-Meier survival curves.

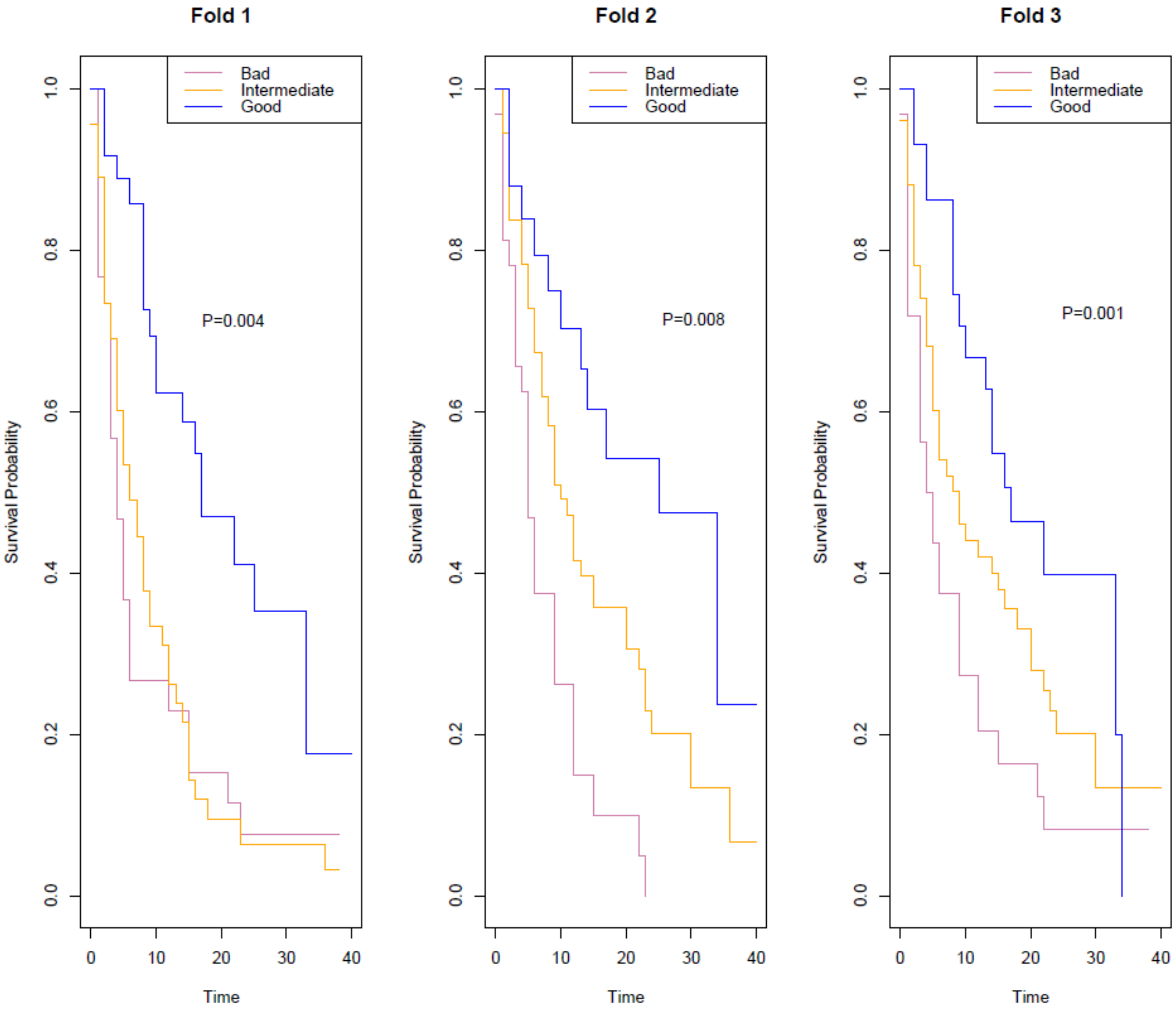

Table S1. Patient Clinical Characteristics.

Clinical Factors	Total SCLC N (%)	ED-SCLC N (%)	Total Missing N (%)
**Patients, n**	183	129	0
**Age, Median (range)**	64 (32-91)	64 (32, 91)	0
≥64	93 (50.8%)	65 (50.4%)	
<64	90 (49.2%)	64 (49.6%)	
**Sex**	183		0
Female	80 (43.7%)	52 (40.3%)	
Male	103 (56.3%)	77 (59.7%)	
**Race**			0
African American/Black	18 (9.8%)	12 (9.3%)	
White	165 (90.2%)	117 (90.7%)	
**BMI, Median (range)**	25.3 (13.7, 56.0)	24.6 (15.5, 45.0)	0
≥25	93 (50.8%)	60 (46.5%)	
<25	90 (49.2%)	69 (53.5%)	
**Marital Status**			7(3.8%)
Married	101 (57.4%)	74 (60.7%)	
Other	38 (21.6%)	24 (19.7%)	
Single	37 (21.0%)	24 (19.7%)	
**Employment Status**			0
Employed	32 (17.5%)	24 (18.6%)	
Not Employed	51 (27.9%)	35 (27.1%)	
Retired	100 (54.6%)	70 (54.3%)	
**Clinical T Stage**			46 (25.1%)
High Stage	59 (43.1%)	46 (45.5%)	
Low Stage	78 (56.9%)	55 (54.5%)	
**Clinical N Stage**			18 (11.8%)
High Stage	129 (78.2%)	108 (85.0%)	
Low Stage	36 (21.8%)	19 (15.0%)	
**Clinical M Stage**			15 (8.2%)
Metastasis	129 (76.8%)	129 (100.0%)	
No Metastasis	39 (23.2%)		
**ICI Type**			0
Anti-PD-L1 Inhibitor	97 (53.0%)	76 (58.9%)	
Combination ICI	15 (8.2%)	12 (9.3%)	
PD-1 Inhibitor	71 (38.8%)	41 (31.8%)	
**Radiation**			0
No	113 (61.7%)	78 (60.5%)	
Yes	70 (38.3%)	51 (39.5%)	
**Surgery**			0
No	152 (83.1%)	112 (86.8%)	
Yes	31 (16.9%)	17 (13.2%)	

Table S2. Summary statistics of patient baseline individual hematological cell types for the full cohort and the metastatic subgroup.

**Hematological Biomarkers**	Total SCLC Median (IQR)	Total SCLC **n (%)**	ED-SCLC Median (IQR)	ED-SCLC **n (%)**	Total Missing n (%)
Eosinophil	0.1(0.05, 0.19)	132	0.08 (0.04, 0.18)	92	51 (27.9%)
Lymphocytes	1.18 (0.77, 1.80)	178	1.30 (0.77, 1.93)	126	5 (2.7%)
Monocytes	0.72 (0.51, 0.95)	178	0.74 (0.51, 0.98)	126	5 (2.7%)
Platelets	278 (195, 363)	178	267 (199, 361)	126	5 (2.7%)
WBC	8.35 (5.73, 11.26)	178	8.57 (6.04, 11.42)	126	5 (2.7%)
Neutrophils	6.10 (3.73, 8.19)	170	6.27 (3.78, 8.58)	123	13 (7.1%)

Table S3. Logistic regression results showing the association of clinical factors with patient response to ICI therapy in SCLC.

**Clinical Characteristic**	**Univariate logistic regression**		**Multivariate logistic regression**	
	**OR (95% CI)**	**p-value**	**OR (95% CI)**	**p-value**
**Age**				
< 64	—			
≥ 64	1.52 (0.52, 4.45)	0.45		
**Sex**				
Female	—			
Male	0.49 (0.17, 1.45)	0.20		
**Race**				
African American/Black	1.45 (0.30, 7.02)	0.64		
White	—			
**Marital Status**				
Married	—	0.42		
Other	0.77 (0.20, 2.97)			
Single	0.25 (0.03, 2.03)			
**Employment Status**				
Employed	—	0.15		
Not Employed	1.26 (0.11, 14.55)			

Clinical Characteristic	Univariate logistic regression		Multivariate logistic regression	
	**OR (95% CI)**	**p-value**	**OR (95% CI)**	**p-value**
Retired	4.28 (0.53, 34.2)			
**BMI**				
< 25	—			
≥ 25	0.84 (0.29, 2.43)	0.75		
**Clinical T Stage**				
High Stage	—			
Low Stage	2.44 (0.63, 9.42)	0.20		
**Clinical N Stage**				
High Stage	—		—	
Low Stage	8.93 (2.51, 31.74)	**<0.001**	3.65 (0.85, 15.65)	**0.08**
**Clinical M Stage**				
Metastasis	—		—	
No Metastasis	14.48 (3.75, 55.97)	**<0.001**	7.82 (1.73, 35.43)	**0.008**
**Radiation**				
No	—			
Yes	0.22 (0.05, 1.02)	0.054		
**Surgery**				
No				
Yes	13.91 (4.33, 44.66)	**<0.001**	9.06 (2.17, 37.86)	**0.003**
**ICI Type**				
Anti-PD1/PD-L1 Inhibitor	0.55 (0.11, 2.70)	0.46		
Combination ICI	—			
**Absolute Eosinophil Count**				
High	—			
Low	1.04 (0.95, 1.15)	0.39		
**Albumin (g/dL)**				

Clinical Characteristic	Univariate logistic regression		Multivariate logistic regression	
	OR (95% CI)	p-value	OR (95% CI)	p-value
High	—			
Low	0.93 (0.86, 1.00)	0.057		
**LDH (U/L)**				
High	—			
Low	0.91 (0.73, 1.13)	0.41		
**NLR**				
High	—			
Low	0.93 (0.86, 1.01)	0.10		
**dNLR**				
High	1.68 (0.52, 5.38)	0.38		
Low	—			
**LMR**				
High	0.72 (0.24, 2.18)	0.56		
Low	—			
**PLR**				
High	—			
Low	1.02 (0.94, 1.11)	0.62		
**SII**				
High	—			
Low	0.98 (0.90, 1.06)	0.59		
**SIRI**				
High	—			
Low	0.98 (0.90, 1.07)	0.65		

Table S4. Univariate Cox proportional hazard regression testing the association of each individual cell types of hematological biomarkers and SCLC patient OS.

**Clinical Characteristics**	**Univariate Cox model**				
	**Total**	**Event**	**HR (95% CI)**	**p-value**	**Cohort**
Eosinophils	178	138	0.57 (0.16, 1.97)	0.37	Full SCLC cohort
Platelets	178	138	1.0 (0.99, 1.00)	0.21	
WBC	178	138	1.0 (0.98, 1.02)	0.97	
Lymphocytes	178	138	0.94 (0.75, 1.17)	0.56	
Neutrophils	170	130	1.0 (0.98, 1.02)	0.94	
Monocytes	178	138	1.0 (0.72, 1.47)	0.87	
Eosinophils	126	101	0.62 (0.19, 2.0)	0.43	ED_SCLC
Platelets	126	101	1.0 (0.99, 1.00)	0.55	
WBC	126	101	1.04 (1.00, 1.08)	0.04	
Lymphocytes	126	101	0.99 (0.77, 1.29)	0.96	
Neutrophils	123	98	1.06 (1.01, 1.10)	0.02	
Monocytes	126	101	1.14 (0.83, 1.58)	0.42	

.
